# miR-18a-5p derived from mesenchymal stem cells-extracellular vesicles inhibits ovarian cancer cell proliferation, migration, invasion, and chemotherapy resistance

**DOI:** 10.1186/s12967-022-03422-7

**Published:** 2022-06-07

**Authors:** Xiaoying Wang, Lili Jiang, Qifang Liu

**Affiliations:** Department of Obstetrics and Gynecology, Shengjing Hospital of China Medical University, 36 Sanhao Street, Heping District, Shenyang, 110004 Liaoning China

**Keywords:** Ovarian cancer, Mesenchymal stem cells, Extracellular vesicles, NACC1, AKT/mTOR, Drug resistance

## Abstract

**Objective:**

Ovarian cancer (OC) is a major threat to women’s health. Mesenchymal stem cells (MSCs) are key regulators in cellular communication by secreting extracellular vesicles (EVs) that are involved in OC. This study probed into the mechanism of human MSCs derived-EVs (hMSC-EVs) in regulating OC cell growth and chemotherapy resistance.

**Methods:**

hMSCs and EVs were isolated and identified. After adding EVs, the uptake of EVs by OC CAOV3/ES2 cells (for in vitro studies), and cell proliferation, migration, and invasion were detected. Downregulated miRNAs in hMSC-EVs were screened and miR-18a-5p expression in OC patients was detected. The prognosis of OC patients was analyzed. Binding sites of miR-18a-5p and NACC1 were predicted and validated. NACC1 expression in OC tissues was measured by RT-qPCR, and its correlation with miR-18a-5p was analyzed by Pearson method. AKT/mTOR pathway activation was assessed by WB. The cisplatin sensitivity of EVs-treated CAOV3 cells was evaluated via MTT assay and tested by tumor formation assay in nude mice.

**Results:**

hMSC-EVs suppressed OC cell proliferation, migration, and invasion. miR-18a-5p was downregulated in OC and miR-18a-5p low expression was associated with a poor prognosis. EV-encapsulated miR-18a-5p targeted NACC1. NACC1 was upregulated in OC tissues. miR-18a-5p knockdown and NACC1 overexpression both annulled the inhibition of hMSC-EVs on OC cell growth. AKT and mTOR were elevated in OC and NACC1 activated the AKT/mTOR pathway in OC cells. hMSC-EVs promoted cisplatin sensitivity of OC cells by carrying miR-18a-5p.

**Conclusion:**

hMSC-EVs-derived miR-18a-5p inhibits OC cell proliferation, migration, invasion, and chemotherapy resistance.

**Supplementary Information:**

The online version contains supplementary material available at 10.1186/s12967-022-03422-7.

## Introduction

Ovarian cancer (OC) is most responsible for death from gynecologic diseases [1], which is more than just a disease, but actually contains a group of tumor types with distinct histology [2]. The mortality of advanced OC patients is up to 70% [3]. OC develops as a consequence of complex interactions of insufficient reliable early diagnosis, high incidence of chemotherapy resistance-induced disease recurrence and heterogeneous tumors [4]. Main clinical treatments for OC include tumor resection surgery and platinum-based chemotherapy [5]. However, in most cases, women with OC are not diagnosed until an advanced stage, and when the cancer has entered the abdominal cavity, it is difficult to be removed surgically, and may develop chemotherapy resistance [6–8]. Hence, exploring new biomarkers with potential clinical application is imperative for OC patients.

Extracellular vesicles (EVs) present promising characteristics as biomarkers for the diagnosis of early cancers [9]. EVs, defined as a heterogeneous group of membrane structures originated from cells, include apoptotic bodies (500–2000 nm), microvesicles (100–1000 nm), and exosomes (30–150 nm), which exist in biological fluids and participate in a variety of biological processes [10, 11]. As critical cell–cell communication medium, EVs are associated with the transmission of intercellular biological signals for the regulation of various pathological processes and cancers, and are closely related to the delivery of anti-OC drugs [12, 13]. In addition, EVs are pivotal players in modulating tumor metastasis, invasion and proliferation [14], which can be generated and released by different cell types, such as mesenchymal stem cells (MSCs) [15]. MSCs are ubiquitous cells in almost all organs; moreover, in different peritoneal cells, MSCs are the cornerstone of cancer spread by participating in the establishment of premetastatic niche and the induction of metastatic and chemoresistant phenotypes [16]. They are pluripotent stromal cells, which can differentiate into a variety of cell types, including osteoblasts, myocytes, and adipocytes, and are easy to obtain from many tissues [17]. MSCs are proposed to own therapeutic functions serving as agent carriers to deliver tumor targeting drug to OC cells [18, 19]. Studies have reported that MSCs have the potential to treat various diseases, such as cancer [20, 21]. Additionally, numerous MSCs-derived exosomes are abundant in tumors [22], hence MSCs have emerged as potential therapeutic targets for diverse malignant tumors, including OC [23]. MSCs and their derived EVs exert antitumor effects in human OC [24]. Bone marrow MSCs participate in the regulation of tumor microenvironment via releasing growth factors, cytokines, and chemokines [25]. MSCs derived-EVs (MSC-EVs) have been evidenced to play an essential role in anticancer therapy [26]. Nevertheless, the mechanism of MSC-EVs in OC is unclear.

MSCs could secrete EVs containing large amounts of microRNAs (miRNAs), mRNAs, and proteins, which could be transported via EVs to target cells, thereby playing a critical role in various cancers [27, 28]. miRNAs, with approximately 22 nucleotides in length, are a range of non-coding and post-transcriptional RNAs that mediate the translation-rate of mRNAs, and have emerged as crucial participants in cancer development [29–31]. Functional miRNAs transferred by MSC-EVs are essential in promoting sensitivity of hepatocellular carcinoma cells [32]. Recently, miR-18a-5p has been reported the involvement in chemotherapy resistance in OC [33]. With regard to mRNAs, nucleus accumbens-associated protein 1 (NACC1) is engaged in cancer pathogenesis and evaluation, such as drug resistance development, cytokinesis promotion and "stem cell-like" phenotypes maintenance [34]. Moreover, NACC1 is aberrantly expressed in OC, which can facilitate OC progression [35]. However, the role of hMSC-EVs-derived miR-18a-5p and NACC1 in OC hasn’t been documented. We hypothesized that hMSC-EVs played underlying roles in OC cell proliferation, migration, invasion, and chemotherapy resistance via releasing miR-18a-5p through NACC1. In this study, we explored the regulatory mechanism of hMSCs-EVs-miRNA-mRNA network in OC, with the aim to offer some novel targets against OC progression.

## Materials and methods

### Ethics statement

This study was authorized by the ethics committee of Shengjing Hospital of China Medical University. All patients have signed the informed consent. We made significant efforts to minimize both the number of animals and their pains.

### Bioinformatics analysis

The differentially expressed miRNAs in hMSC-EVs were predicted via the GEO (https://www.ncbi.nlm.nih.gov/geo/) and EVmiRNA (http://bioinfo.life.hust.edu.cn/EVmiRNA#!/) databases. The downstream targets of miRNAs were anticipated through Starbase (http://starbase.sysu.edu.cn/), TargetScan (http://www.targetscan.org/vert_71/), and GEPIA (http://gepia2.cancer-pku.cn/#index) databases. The significantly differentially expressed genes in OC samples and normal samples in TCGA and GTEx were downloaded.

### Clinical sample collection

This study separately collected tissues of 76 patients and serum samples of 18 patients who were hospitalized in Shengjing Hospital of China Medical University and underwent surgery for OC from January 2018 to December 2021. Inclusion criteria were: (1) good preoperative general conditions, and no preoperative history of adjuvant chemo-radiotherapy; (2) clear diagnosis of OC, operative indication and no obvious operation contraindication; (3) confirmation of ovarian malignant tumor by postoperative routine pathology diagnosis; (4) complete clinic-pathological data and follow-up data. Exclusion criteria were: (1) diagnosis of ovarian malignant tumor complicated with severe cardiopulmonary insufficiency, cardiovascular and cerebrovascular diseases, liver and kidney failure, and other basic diseases that seriously affected the survival of patients after hospitalization, and preoperative history of neoadjuvant chemotherapy; (2) unclear diagnosis of OC, diagnosis of benign tumor, recurrent tumor, complicated with other tumors, and loss of surgical opportunity for late metastasis; (3) confirmation of other tumors, rather than ovarian malignant tumor by postoperative routine pathology diagnosis; (4) lost clinic-pathological data and no follow-up data due to various reasons. Meanwhile, we collected tissues from 36 patients with benign OC and serum samples from 20 healthy controls. Survived patients, died patients, and overall survival time were recorded during the 24-month follow-up, and the survival curve was analyzed.

### hMSCs and OC cells culturing

OC CAOV3/ES2 cells and normal ovarian epithelial cells IOSE80 [36] (Cell Bank of Chinese Academy of Sciences, Shanghai, China) were cultured in a RPMI-1640 cell culture medium (C11875500BT, Gibco, Grand Island, NY, USA) including 10% fetal bovine serum (FBS) (Gibco, C11108862) and 1% penicillin/streptomycin (Thermo Fisher, Waltham, MA, USA, 10378016). hMSCs (PromoCell, Heidelberg, Germany) were cultured in a Mesenchymal Stem Cell Growth Medium (Promocell). Upon 80% confluence, cells were routinely detached using 0.25% trypsin for 3–5 min. When cells were observed to become round and their intercellular space was enlarged under a microscope, the trypsin was discarded and cells were dispersed in a fresh culture medium. Cells were centrifuged and then the medium was removed. After that, cells were passaged at 1:3 or 1:2. All cells were cultured at 37 °C in the incubator containing 5% CO_2_.

### Characterization of hMSCs

hMSCs were detached, centrifuged, and washed with phosphate-buffered saline (PBS), and then resuspended in the Stain Buffer and counted. The cell suspension was transferred to new Eppendorf tubes (1.5 mL), with about 5 × 10^4^ cells in each tube. According to the concentrations of antibodies recommended in the instructions of flow cytometry, 5 μL CD29 (ab263847, Abcam, Cambridge, UK), CD34 (ab81289, Abcam), CD45 (ab40763, Abcam), CD73 (ab202122, Abcam), CD90 (ab23894, Abcam), CD105 (ab2529, Abcam), CD117 (ab45924, Abcam), human leukocyte antigen-D-related (HLA-DR; ab92511, Abcam) antibodies and isotype controls were added to 50 μL cell suspension respectively. After being mixed evenly, the cell suspension containing antibodies was incubated for 30 min in a refrigerator at 4 °C in the dark, washed 3 times with pre-cooled Stain Buffer and centrifuged for 5 min at 300*g*. Then unbound antibodies were washed away. Finally, cells were resuspended in flow tubes with 500 μL Stain Buffer and detected by flow cytometry, and analyzed and processed using Flowjo 7.6 software.

OriCell^™^ hMSC osteogenic differentiation kit, OriCell^™^ hMSC adipogenic differentiation kit, and OriCell^™^ hMSC chondrogenic differentiation kit were from Cyagen Biosciences (Guangzhou, China). The specific experiment operation was conducted following the instructions of the manufacturer’s kit.

### Extraction, identification, and grouping of EVs

Cell supernatant (10 mL, obtained from 5 × 10^6^ cells after 48-h culture) was centrifuged for 10 min at 300*g* at 4 °C, then centrifuged at 2000*g* at 4 °C for 10 min, centrifuged at 10,000*g* at 4 °C for 30 min and then collected. After that, the supernatant was filtered into an ultracentrifuge tube through a 0.22 μm filter, and ultra-centrifuged in a Type 90 Ti rotor (Beckman, Palo Alto, CA, USA) at 100,000*g* for 70 min at 4 °C, and then the supernatant was discarded. The precipitation was resuspended by adding 1 mL PBS into the ultracentrifuge tube, and the Type 90 Ti rotor (Beckman) was used again for centrifugation at 100,000*g* for 70 min at 4 °C. The supernatant was removed and the EVs precipitation was collected. Protein concentration of EVs was determined using the bicinchoninic acid (BCA) detection reagent (ComWin Biotech, Beijing, China). EVs were transfected into a complete cell culture medium, and the protein concentration of EVs was adjusted to 5 μg/mL. The PBS was used as the control group.

The collected precipitation was resuspended with 50 μL PBS, and the suspension was placed on the copper grids for 20 min at room temperature. Subsequently, the suspension was washed with PBS for 5 min × 3 times, fixed in 1% glutaraldehyde for 5 min, and rinsed with double distilled water for 2 min × 10 times. Thereafter, 4% acetic acid uranium dioxygen was added for negative staining for 5 min. Filter paper was used to absorb residual liquid along the outside of the copper grids, followed by drying. Subsequently, the EVs were observed and photographed using the Tecnai G2 transmission electron microscopy (TEM, FEI Company, Hillsboro, OR, USA). The separated EVs were diluted 200 to 600 times for the particle size analysis using Nanosight instrument (Malvern Panalytical, Malvern, Worcestershire, UK). EVs were identified using Western blot (WB) analysis after being lysed using radioimmunoprecipitation assay (RIPA) lysate.

The EVs used in this study were named as: EVs, EVs^−NC^ (EVs separated and extracted after inhibitor NC transfection into hMSCs), and EVs^−inhi^ (EVs isolated and extracted after miR-18a-5p inhibitor transfection into hMSCs).

### Uptake of EVs

The uptake of EVs by CAOV3/ES2 cells was observed by PKH26 staining. Cell coverslips were pre-placed on 24-well plates. CAOV3/ES2 cell suspension was seeded in 24-well plates at 5 × 10^4^ cells/well. After the cells adhered to the well wall, the pre-stained EVs suspension was attached to the well plate at a protein concentration of 80 μg/mL followed by co-culture for 24 h. The cell coverslips were fixed with 4% paraformaldehyde in the dark for 20 min, stained with 4'-6-diamino-2-phenylindole, and then blocked with anti-fluorescent quenching agent. Later, the uptake of EVs was observed under a confocal fluorescence microscope.

### Cell treatment and grouping

CAOV3/ES2 cells were assigned into the following 9 groups: blank group (without any treatment, normal culture), PBS group (treated with EVs solvent PBS for 12 h), EVs group (treated with 20 μg EVs for 12 h), EVs^−NC^ group (treated with 20 μg EVs^−NC^ for 12 h), EVs^−inhi^ group (treated with 20 μg EVs^−inhi^ for 12 h), mimics NC group (transfected with mimics NC for 48 h), miR-18a-5p mimics group (transfected with miR-18a-5p mimics for 48 h), and EVs + oe-NACC1 group (treated with 20 μg EVs for 12 h after transfection with overexpressed NACC1 plasmid pcDNA3.1-NACC1 for 48 h), and EVs + oe-NC (supplemented with 20 μg EVs for 12 h after transfection with pcDNA3.1-NC for 48 h). The miR-18a-5p mimics, mimics NC, pcDNA3.1-NACC1, and pcDNA3.1-NC were provided by GenePharma (Shanghai, China). Briefly, the cells were subjected to transfection for 48 h using Lipofectamine2000 (Invitrogen, Carlsbad, CA, USA).

### 3-(4,5-dimethylthiazol-2-yl)-2,5-diphenyltetrazoliumbromide (MTT) assay

MTT solution (20 μL) (5 mg/mL, Sigma-Aldrich, St Louis, MO, USA) was added into each well for color rendering after 1, 2, 3, 4, 5, and 6-day culturing respectively, followed by further 4-h incubation with 5% CO_2_ at 37 °C. Next, the culture medium was discarded. Each well was supplemented with 150 μL dimethyl sulphoxide, shaking gently for 10 min to promote the dissolution of the crystals. The optical density (OD) value at 495 nm (OD_495_ value) of each well was measured on the enzyme-linked immunoassay instrument.

### Transwell assays

Transwell chamber (140,644, Thermo Fisher) was placed into 24-well microplates, with the basolateral chamber covered with 500 mL medium containing 20% FBS. The 24-well microplates were incubated for 2 h. Cells were detached using trypsin, washed in PBS or serum-free medium 3 times, resuspended and counted, and cell concentration was made to 2 × 10^5^ cells/mL. After dispersing and mixing, 500 mL cell suspension was put to the apical chamber. There should be no bubbles between the apical and basolateral chambers. The apical chamber was added with Matrigel to perform the invasion assay and contained no Matrigel in the migration assay. The 24-well microplates were placed in a cell incubator for further culturing. After 20 h, the Transwell chamber was taken out, and the apical chamber was drained of the liquid, washed with PBS, and moved to the wells prefilled with about 800 mL methanol or paraformaldehyde for 30-min fixing. After that, the chamber was washed twice using PBS and stained using 800 μL crystal violet staining solution for 15–30 min away from light, followed by gentle washes in double distilled water to wash away the staining solution. The liquid in the apical chamber was discarded. Cells on the membrane surface at the bottom of the apical chamber were carefully cleaned using a wet cotton swab, then 10 fields of vision were randomly selected under the microscope (× 20) for photographing, and the cells in each photograph was counted, with the average number for statistical analysis.

### Reverse transcription quantitative polymerase chain reaction (RT-qPCR)

Total RNA was extracted by TRIzol (15,596,026, Thermo Fisher) and then reversely transcribed into cDNA using PrimeScript RT reagent kits (Takara, Otsu, Shiga, Japan). TaqMan primers and probes were provided by Takara. qPCR was performed using the ABI PRISM 7900 sequence detection system of SYBR Green II (Takara) under the following reaction conditions: pre-denaturation at 95 °C for 10 min, and then 40 cycles of denaturation at 95 °C for 10 s, annealing at 60 °C for 20 s, and finally extension at 72 °C for 34 s. GAPDH and U6 acted as internal references. Data were evaluated using 2^−ΔΔCt^ method. The amplified primer sequences are listed in Table 1.

**Table 1:** Primer sequences

Gene	Forward 5ʹ–3ʹ	Reverse 5ʹ–3ʹ
miR-18a-5p	GGGATGAGATGAAGCACT	TGCGTGTCGTGGAGT
NACC1	TTTCAAACAAAGATGCCACA	GTTCCCTAAACTCCTAAGCAGATA
AKT	CCCTGAGGCATTTAGGCAGCTA	AGGTAGAGAGGTGGCTTAGGCT
mTOR	CAGCCAGATGCAATCAATGCC	TCTGCTCCTGAGCATTGACGTC
U6	CTCGCTTCGGCAGCACATATACT	ACGCTTCACGAATTTGCGTGTC
GAPDH	GTCGATGGCTAGTCGTAGCATCGAT	TGCTAGCTGGCATGCCCGATCGATC

### Dual-luciferase reporter assay

The binding sites of miR-18a-5p and NACC1 were predicted by Starbase (http://starbase.sysu.edu.cn/index.php). The complementary binding sequences of miR-18a-5p and NACC1 and their mutation sequences were amplified and cloned into the pmiR-GLO luciferase vector (Promega, Madison, WI, USA) to construct the pGL-NACC1-wild type (WT) plasmid and the corresponding pGL-NACC1-mutant (MUT) plasmid. Subsequently, the plasmids were respectively co-transfected with mimic NC or miR-18a-5p mimic (GenePharma) into HEK293T cells using Lipofectamine 2000 (Invitrogen, Carlsbad, CA, USA). After 48 h, luciferase activity was determined.

### WB

Cells to be tested were lysed using RIPA lysate containing protease inhibitor (Boster, Wuhan, China). Cells were centrifuged at 12,000 rpm for 10 min at 4 °C. The supernatant was absorbed, and the protein concentration was measured using the BCA detection reagent (CW0014, ComWin Biotech). Electrophoresis was performed at 60 V, and 120 V was used after proteins entering the separating gel. After electrophoresis, the proteins were transferred onto polyvinylidene fluoride (PVDF) membranes using the wet transfer method in a cold chamber at 4 °C for 2 h. Thereafter, PVDF membranes were removed, blocked with 5% skim milk-Tris-buffered saline-Tween 20 (TBST), and incubated for 1–2 h. Next, the membranes were placed in an incubator, incubated overnight with primary antibodies (Table 2) at 4 °C, and washed with TBST for 10 min × 3 times. Later, the membranes were probed 1 h with goat anti-rabbit immuroglobulin G labeled with horseradish peroxidase (1:5000, ComWin) followed by washing with TBST for 10 min × 3 times. Afterwards, chemiluminescence, X-ray tablet pressing, developing and fixing were performed. The data were analyzed with GAPDH as the control.

**Table 2:** Antibody information

Name	Catalog number	Dilution rate
NACC1	BF0187	WB (1:1000)
p-AKT	Abcam ab38449	WB (1:1000)
AKT	Abcam ab8805	WB (1:500)
p-mTOR	Abcam ab109268	WB (1:2000)
mTOR	Abcam ab134903	WB (1:10000)
GAPDH	Abcam ab9485	WB (1:1000)
CD63	Abcam ab134045	WB (1:1000)
CD9	CST 13403	WB (1:1000)
CD81	Thermo MA5-17939	WB (1:1000)
HSP70	Abcam ab2787	WB (1:1000)
Calnexin	CTS 2679	WB (1:1000)

### Determination of drug resistance

CAOV3 cells in the exponential phase were added with cisplatin at a gradient concentration, with an initial concentration of 0.05 μg/mL. After 3 stable passages, cells were supplemented with an increasing concentration of 0.05 μg/mL until the cells could be stably grown and passaged in 1 μg/mL cisplatin. Afterwards, the cells were treated with 2 μg/mL cisplatin every 3 days for 24 h until the cells could be stably grown and passaged in 2 μg/mL cisplatin. The induction time was 30 weeks to construct CAOV3 drug-resistant cell line. Later, the effect of different cisplatin concentrations (0.0, 0.1, 0.5, 1.0, 2.0, 4.0, 6.0, 8.0, and 10.0 μM) on the viability of resistant cells was detected by MTT assay and the IC_50_ value of cisplatin in OC drug-resistant cell line CAOV3 was calculated.

### Colony formation assay

OC drug-resistant cells CAOV3 in the exponential phase were detached into single-cell suspension with trypsin. Cells were diluted to 1 × 10^5^ cells/mL. Each well of the 6-well microplates was seeded with 200 µL cell suspension, followed by the addition of culture medium to 2 mL. Then cells were incubated in RPMI-1640 containing 10% FBS and cisplatin at the concentration of IC_50_ for 2 weeks, with the liquid changed regularly. When visible cell clones appeared in the cells, the supernatant was discarded, and cells were fixed in 4% paraformaldehyde for 15 min and stained with 0.1% crystal violet. Finally cell clones were counted.

### Xenograft tumors in nude mice

OC parental cells and drug-resistant cell line CAOV3 were detached into cell suspension, which were then diluted to a concentration of 10^5^ cells/mL using the complete growth medium. Cell suspension (0.4 mL) from each sample was mixed with an equal amount of matrigel. Mice were anesthetized, and the lower abdomen was disinfected, and then 0.2 mL cells/matrigel mixture was immediately subcutaneously injected into the right lower abdomen of mice in the corresponding group using sterile syringe until the formation of pimple. After injection of 6 mice in each group, mice were put back into cages for further feeding. From the 7^th^ day after inoculation, the tumor volume was measured every 2 days according to the formula (tumor volume [mm^3^] = ab^2^/2) (a, long diameter of the tumor; b, short diameter of the tumor). The mice injected with drug-resistant cell line CAOV3 were treated with cisplatin and EVs. Cisplatin was prepared to 25 mg/kg stock solution and administered intraperitoneally based on the weight of mice (0.1 mL/10 g), while 20 μg EVs were injected at the same time, once every 2 days [37–40]. Mice were euthanized with an intraperitoneal injection of excessive pentobarbital sodium on the 19^th^ day and tumors were weighed.

### Hematoxylin–eosin (HE) staining

The lung tissues were collected from nude mice, fixed with 10% neutral formalin, embedded in paraffin, and dewaxed in xylene. The tumor sections were stained with hematoxylin, washed with distilled water, and soaked in 95% ethanol, followed by eosin staining. Later, the sections were hydrated with a certain gradient of ethanol, dehydrated with xylene, dried, and fixed with neutral resin. Thereafter, the pathological changes of lung tissues and the metastasis of tumor nodules were observed under an optical microscope.

### Statistical analysis

The results were described as mean ± standard deviation (SD). The independent sample *t* test was adopted for data comparison between two groups, and one-way or two-way analysis of variance (ANOVA) was adopted for comparison between multiple groups. SPSS 17.0 (SPSS, Inc, Chicago, IL, USA) and GraphPad Prism 8.0 (GraphPad Software, San Diego, CA, USA) were used for data statistical analysis. The *p* < 0.05 was regarded statistically significant.

## Results

### Characterization of hMSCs and EVs

**Fig. 1:**
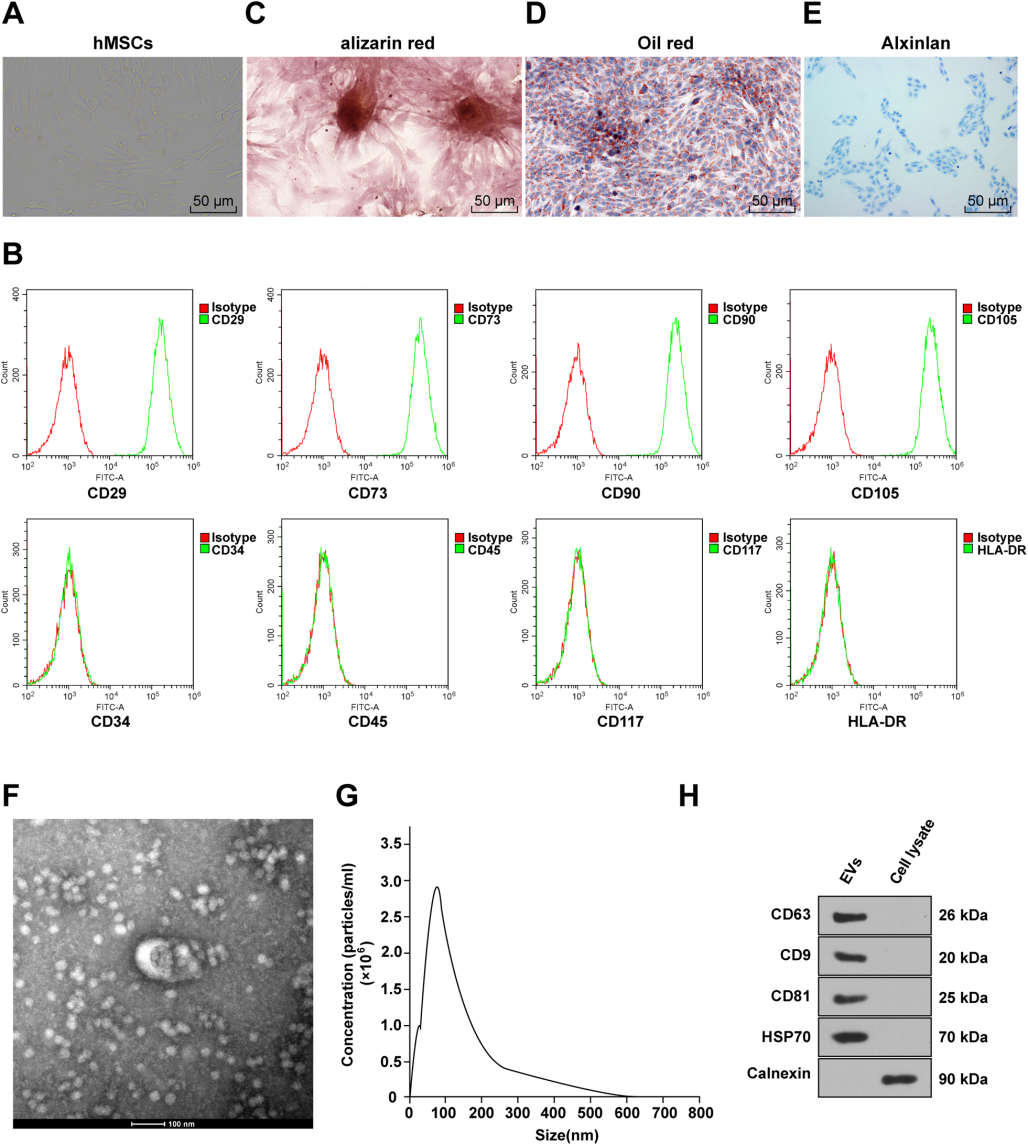
Identification of hMSCs and EVs. The hMSCs were passage-cultured. **A** Morphology of the fourth-passage hMSCs was observed under a microscope; **B** Expression of specific molecular markers CD29, CD34, CD45, CD73, CD90, CDl05, CD117 and HLA-DR was detected using flow cytometry; **C** Osteogenic differentiation was observed by Alizarin red staining; **D** Adipogenic differentiation was observed via Oil Red O staining; **E** Cartilage differentiation was observed through Alcian blue; **F** Morphology of hMSC-EVs was observed using TEM after the extraction of EVs; **G** Size distribution of EVs was analyzed by NTA; **H** Expression of CD63, CD9, CD81, HSP70 and Calnexin in EVs was measured using WB

The relatively uniform and viable hMSCs were obtained after 3 to 4 passages of subculture, with homogeneous cell morphology, a typical shuttle shape, and arranged in whorls (Fig. 1A). The surface antigens of hMSCs were characterized using flow cytometry, which demonstrated upregulated hematopoietic stem cell markers CD29, CD73, CD90, and CDl05, but lowly or not expressed CD34, CD45, CD117, and HLA-DR (Fig. 1B). In addition, hMSCs differentiated into osteoblasts after the addition of osteogenic culture induced liquid. On the 14th day, the entire cells were filled with calcium particles, the cells grew in colonies, the central cells gradually fused, and a large number of calcium nodules were suspended in the cell surface and stained red with Alizarin red (Fig. 1C), indicating the potential of hMSCs to differentiate into osteoblasts. After adipogenic differentiation induction of hMSCs, the deposition of lipid components was verified by Oil Red O staining, which revealed the adipogenic differentiation ability of hMSCs (Fig. 1D). Furthermore, after 28-day chondrogenic differentiation induction of hMSCs, hMSCs formed a chondrosphere, and the internal acidic mucopolysaccharide in cartilage tissues could be stained light blue by Alcian blue (Fig. 1E), illustrating that hMSCs had the ability to differentiate into chondrocytes.

Subsequently, the culture supernatant of hMSCs was collected, and EVs produced by hMSCs were extracted by differential centrifugation. The protein concentration of EVs in every 10 mL cell supernatant was determined to be 0.56 ± 0.07 mg/mL. The morphology and size of collected EVs were observed by TEM, and we found that the size of EVs was mainly between 50 and 150 nm, along with oval characteristics (Fig. 1F). Nanoparticle tracking analysis (NTA) revealed that the particle size of EVs was mainly concentrated around 90 nm, with a concentration of 2.7 × 10^6^ cells/mL (Fig. 1G). WB detected the positive expression of the EV markers CD63, CD9, CD81 and HSP70, but negative expression of the endoplasmic reticulum marker Calnexin (Fig. 1H). The above results elicited the successful isolation of normal hMSC-EVs.

### hMSC-EVs suppressed OC cell proliferation, migration, and invasion

**Fig. 2:**
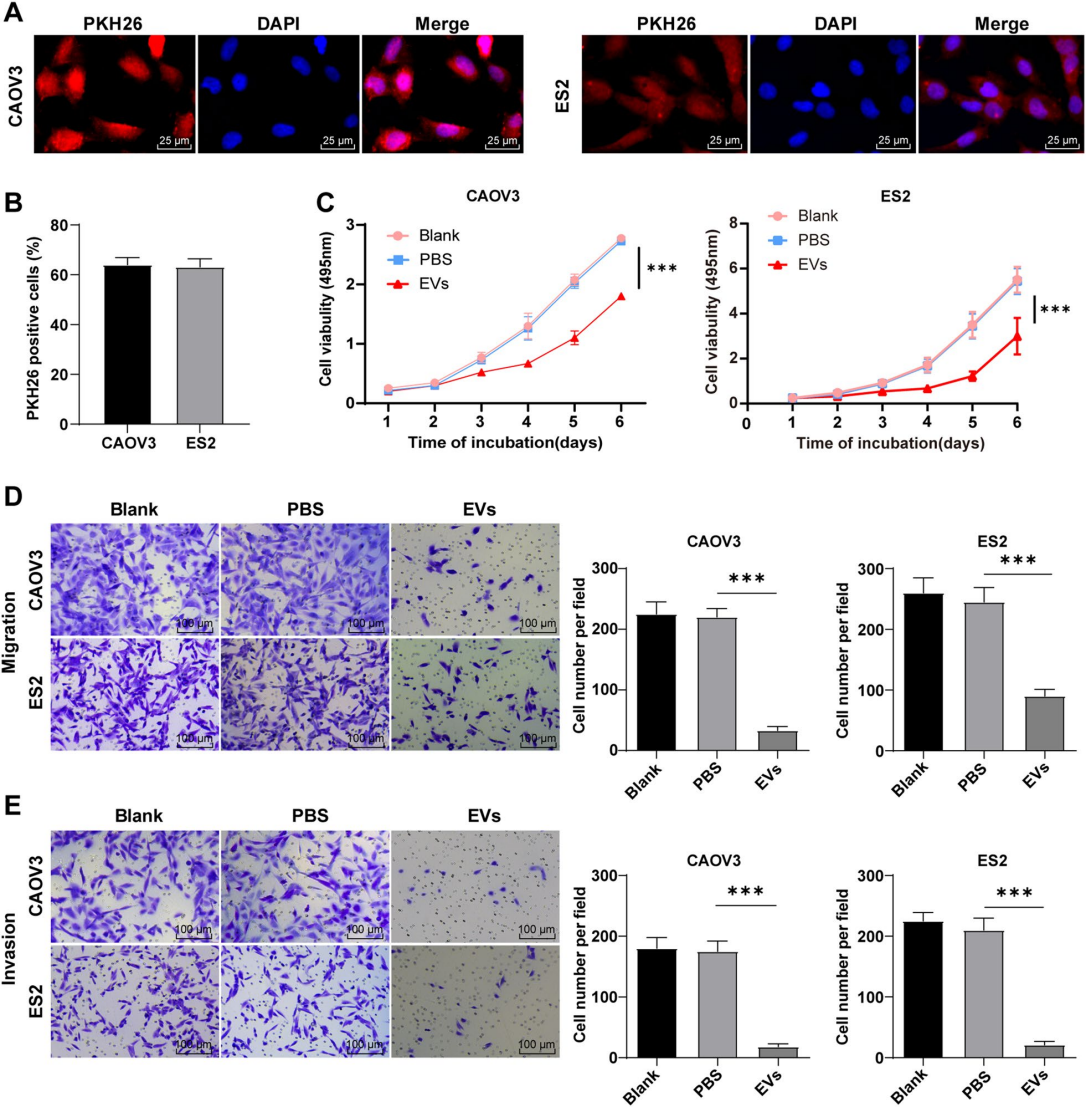
hMSC-EVs suppressed OC cell proliferation, migration, and invasion. OC CAOV3/ES2 cells were treated with hMSC-EVs. **A**, **B** Immunofluorescence assay was used to detect the uptake of EVs by OC cells; **C** MTT assay was performed to detect the cell viability; **D**, **E** Transwell assays were employed to analyze OC cell migration and invasion. Cell experiments were repeated 3 times. Data were expressed as mean ± SD and processed by two-way (panel C) or one-way ANOVA (panels D/E). Tukey's multiple comparisons test was adopted for the post hoc test. ***P < 0.001

To explore the role of hMSC-EVs in OC, the OC CAOV3/ES2 cells were selected for in vitro studies. First, the uptake experiment found that EVs could be internalized by CAOV3/ES2 cells (Fig. 2A, B). Subsequently, MTT assay was used to assess the effect of EVs on the proliferation of OC cells, which showed that EVs significantly repressed OC cell proliferation compared with the PBS group (*P* < 0.05) (Fig. 2C). Next, Transwell assays revealed that hMSC-EVs evidently reduced OC cell migration and invasion compared with the PBS group (all *P* < 0.05) (Fig. 2D, E). Generally, hMSC-EVs inhibited OC cell proliferation, migration, and invasion.

### miR-18a-5p was downregulated in OC and associated with a poor prognosis

**Fig. 3:**
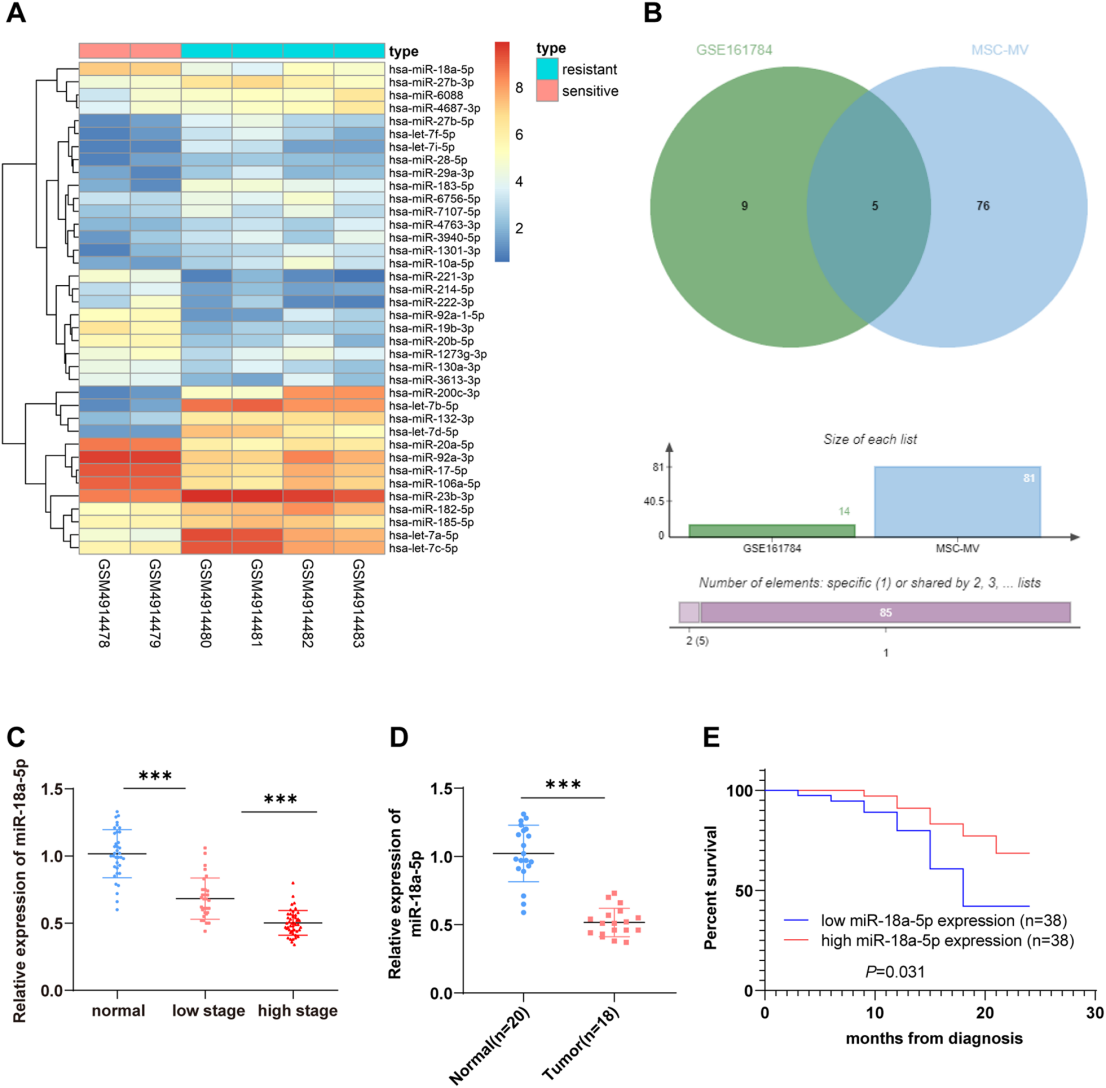
miR-18a-5p was downregulated in OC. **A** Heatmap of significantly downregulated miRNAs in OC microarray GSE161784 (abscissa: sample name; ordinate: miRNA name; small square: the expression level of a miRNA in a sample; upper right histogram: color scale); **B** Intersection of significantly reduced miRNAs in chemotherapy-resistant samples and miRNAs expressed in hMSC-EVs in the EVmiRNA database was obtained, with the middle as the intersection; **C**, **D** Expression of miR-18a-5p was determined by RT-qPCR; **E** Prognosis of OC patients with low and high miR-18a-5p expression was assessed using Kaplan–Meier survival analysis. Data analysis was performed using one-way ANOVA (panel C) with Tukey's multiple comparisons test for the post hoc test. The independent sample *t* test was used for comparisons between the two groups (panel D). ***P < 0.001

To further investigate the miRNA that is closely related to OC development, a cisplatin-resistant OC expression microarray GSE161784 was obtained through the GEO database. After differential analysis of miRNA expression in this microarray, we identified 38 markedly differentially expressed miRNAs (Fig. 3A), of which 14 miRNAs were downregulated in cisplatin-resistant samples. Additionally, miRNAs expressed in hMSC-EVs were identified through the EVmiRNA database (Additional file 1: Table S1), then the intersection of above two groups of data was taken (Fig. 3B), and finally 5 candidate miRNAs were acquired (Additional file 2: Table S2). Among these 5 candidate miRNAs, miR-18a-5p is involved in regulating drug resistance in OC [41].

Subsequently, to analyze miR-18a-5p expression in OC, we collected 76 OC tissue samples (including 30 cases in early OC and 46 cases in advanced OC) and 36 benign ovarian tumor tissue samples. RT-qPCR unveiled reduced miR-18a-5p expression in OC tissues and a correlation with tumor progression (*P* < 0.05) (Fig. 3C). In addition, we examined the circulating level of serum miR-18a-5p in 18 OC patients and 20 healthy controls by RT-qPCR and found that the circulating level of serum miR-18a-5p was significantly lower in OC patients than in healthy controls (*P* < 0.01) (Fig. 3D). At the same time, we divided 76 OC patients into high miR-18a-5p expression group (> 0.545, *n* = 38) and low miR-18a-5p expression group (< 0.545, *n* = 38) base on the median of miR-18a-5p expression (0.545) in tumor tissues. The analysis of patient survival revealed that low miR-18a-5p expression indicated short survival relative to high miR-18a-5p expression in OC patients (*P* < 0.05) (Fig. 3E). In short, miR-18a-5p was weakly expressed in OC and associated with a poor prognosis.

### miR-18a-5p knockdown annulled the inhibition of hMSC-EVs on OC cell proliferation, migration, and invasion

**Fig. 4:**
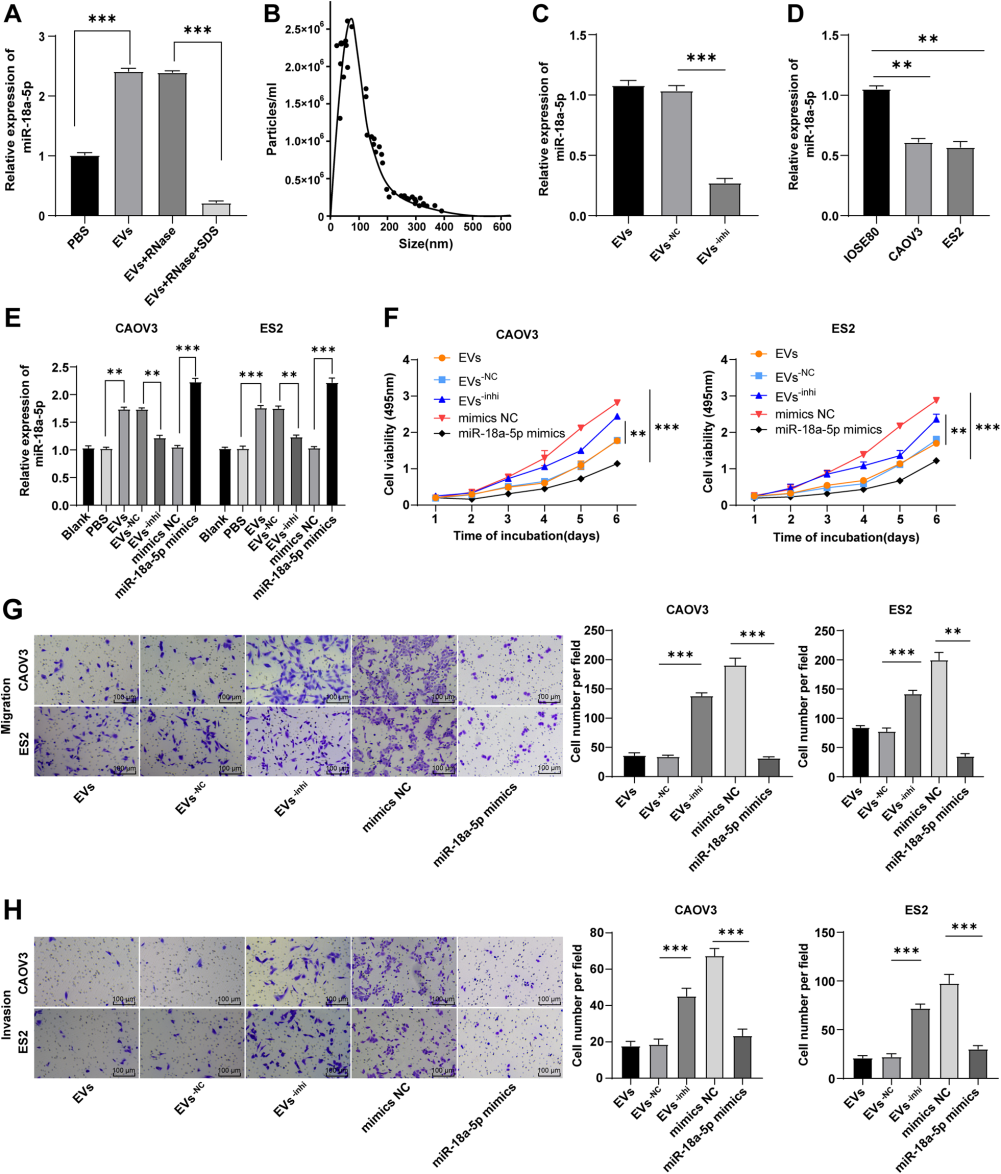
miR-18a-5p knockdown partially annulled the inhibition of EVs on OC cell proliferation, migration and invasion. **A** EVs were treated differently and miR-18a-5p expression in EVs was detected by RT-qPCR; **B** Size distribution of EVs was analyzed by NTA; **C**–**E** miR-18a-5p expression was measured via RT-qPCR; **F** Cell viability was assessed using MTT assay; **G**, **H** Migration and invasion of OC cells were evaluated by Transwell assays. Cell experiments were replicated 3 times. Data were presented as mean ± SD. Data were analyzed by two-way (panel F) or one-way ANOVA (panels A/C/D/E/G/H).One-way ANOVA was employed for comparisons between groups and Tukey's multiple comparisons test was performed for the post hoc test. **P < 0.01, ***P < 0.001

To study whether hMSC-EVs play roles in OC by delivering miR-18a-5p, the expression of miR-18a-5p in EVs was first detected by RT-qPCR, which found that miR-18a-5p in the EVs group was remarkably higher than that in the PBS group. After the further RNase treatment, miR-18a-5p expression in EVs showed no prominent change, while miR-18a-5p was clearly reduced when sodium dodecyl sulfate was further added (all *P* < 0.01) (Fig. 4A), indicating that miR-18a-5p was encapsulated in EVs. Subsequently, hMSCs were transfected with miR-18a-5p inhibitor and EVs were isolated and extracted. NTA assay revealed that particle size of EVs was mainly concentrated around 90 nm at a concentration of 2.68 × 10^6^ cells/mL, and the EV concentration exhibited no evident change compared with that (2.7 × 10^6^ cells/mL) before transfection with miR-18a-5p inhibitor (*P* > 0.05) (Fig. 4B). Additionally, significantly reduced miR-18a-5p was observed in EVs (*P* < 0.01) (Fig. 4C).

Additionally, we detected the expression of miR-18a-5p in OC cells (CAOV3/ES2) and normal ovarian epithelial cells (IOSE80) by RT-qPCR, and the results showed that the relative expression of miR-18a-5p was notably lower in OC cells than in normal cells (*P* < 0.01) (Fig. 4D). Following treatment of EVs^−inhi^ or miR-18a-5p mimics in OC cells, we found that compared with the EVs^−NC^ group, miR-18a-5p was markedly decreased in OC cells in the EVs^−inhi^ group, which was elevated in the miR-18a-5p mimics group (all *P* < 0.01) (Fig. 4E). Next, the proliferation, migration, and invasion of OC cells were detected, and the results demonstrated that after inhibiting miR-18a-5p in EVs, proliferation, migration, and invasion of OC cells were considerably enhanced; after overexpression of miR-18a-5p alone in OC cells, the proliferation, migration, and invasion of OC cells were significantly reduced compared with the mimics NC group (all *P* < 0.01) (Fig. 4F–H). These results indicated that miR-18a-5p overexpression notably restrained OC cell growth and miR-18a-5p silencing in EVs partially reversed the inhibition of EVs on OC cells, which indicated that the inhibitory effect of EVs on OC cells was achieved by transferring miR-18a-5p into the cells.

### miR-18a-5p targeted and inhibited NACC1 expression

**Fig. 5:**
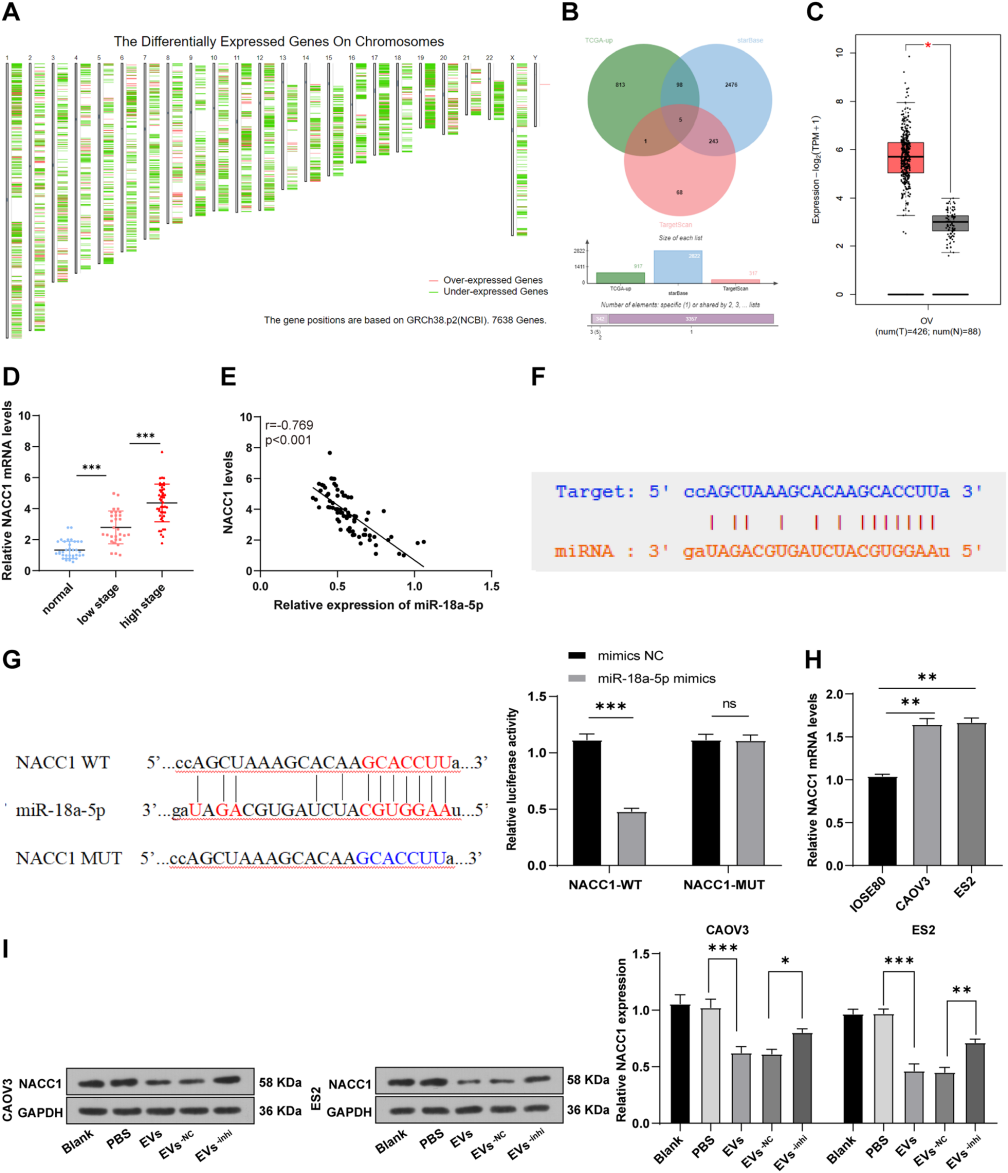
miR-18a-5p targeted and inhibited NACC1 expression. **A** Chromosome expression maps of significantly differentially expressed genes in OC included in TCGA and GTEx (green and red lines: evidently differentially expressed genes; position: the location in chromosome; red: upregulated genes; green: downregulated genes); **B** Prediction of target genes of miR-18a-5p: 3 circles respectively represented the prediction results of Starbase database, TargetScan databases and significantly upregulated genes in OC, and the middle part indicated the intersection of the 3 groups of data; **C** NACC1 expression in OC and normal samples included in TCGA and GTEx (abscissa: sample type; ordinate: expression level; red: tumor sample; gray: normal sample); **D** Expression of NACC1 mRNA in 76 early/advanced OC tissues and 36 benign ovarian tumor tissues was measured by RT-qPCR; **E** Correlation analysis of miR-18a-5p and NACC1; **F** Binding sites of miR-18a-5p and NACC1 was predicted via Starbase database; **G** Targeted binding of miR-18a-5p and NACC1 was validated using dual-luciferase assay; **H** Expression of NACC1 mRNA in CAOV3/ES2 cells and normal ovarian epithelial cells (IOSE80) was detected by RT-qPCR; **I**, NACC1 expression in OC cells was determined by WB. Cell experiments were repeated 3 times. Data were exhibited as mean ± SD. One-way ANOVA was used for comparisons between groups and Tukey's multiple comparisons test was employed for the post hoc test. *P < 0.05, **P < 0.01, ***P < 0.001

The downstream target genes of miR-18a-5p were predicted by Starbase and TargetScan databases to estimate the regulatory mechanism of miR-18a-5p derived from hMSC-EVs on OC cells. Meanwhile, the significantly differentially expressed genes in OC and normal samples included in TCGA and GTEx were identified through GEPIA database (Fig. 5A), then the intersection of significantly upregulated genes in OC and predicted target genes were taken (Fig. 5B), and finally 5 candidate genes were identified. Among these 5 candidate genes, NACC1 had the largest fold of upregulation in OC (Table 3, Fig. 5C), suggesting that NACC1 may have played a more important regulatory role. Furthermore, RT-qPCR assay showed that NACC1 mRNA was clearly upregulated in tissues from 76 OC patients (*P* < 0.01) (Fig. 5D), and it was prominently negatively-correlated with miR-18a-5p (*P* < 0.001) (Fig. 5E), eliciting that miR-18a-5p may regulate OC by targeting NACC1.

**Table 3:** Differential expression of candidate genes

Gene symbol	Log2 (fold change)	adjp
RBBP8	2.030	3.37E-51
PARD6B	2.024	3.92E-83
RTN2	2.328	3.66E-45
TPM3	2.095	5.04E-75
NACC1	2.695	3.65E-68

Furthermore, the binding sites of miR-18a-5p and NACC1 were predicted via Starbase database (Fig. 5F). The luciferase activity of miR-18a-5p mimics and NACC1-WT co-transfected cells was significantly lower than that of mimics NC and NACC1-WT co-transfected cells (*P* < 0.05), while the luciferase activity of NACC1-MUT-transfected cells presented no obvious change (*P* > 0.05) (Fig. 5G), which illustrated the targeted binding of miR-18a-5p and NACC1. Subsequently, we examined NACC1 mRNA expression in CAOV3/ES2 cells and normal ovarian epithelial cells (IOSE80) by RT-qPCR and measured NACC1 level in OC cells treated with EVs from different groups by WB analysis. The results revealed that NACC1 mRNA expression was markedly higher in OC cells than in normal cells (*P* < 0.01) (Fig. 5H). After adding EVs, NACC1 expression was clearly decreased in CAOV3 and ES2 cells, while increased after inhibiting miR-18a-5p in EVs (all *P* < 0.01) (Fig. 5I). To sum up, miR-18a-5p targeted and inhibited NACC1 expression.

### NACC1 overexpression reversed the inhibition of hMSC-EVs on OC cells

**Fig. 6:**
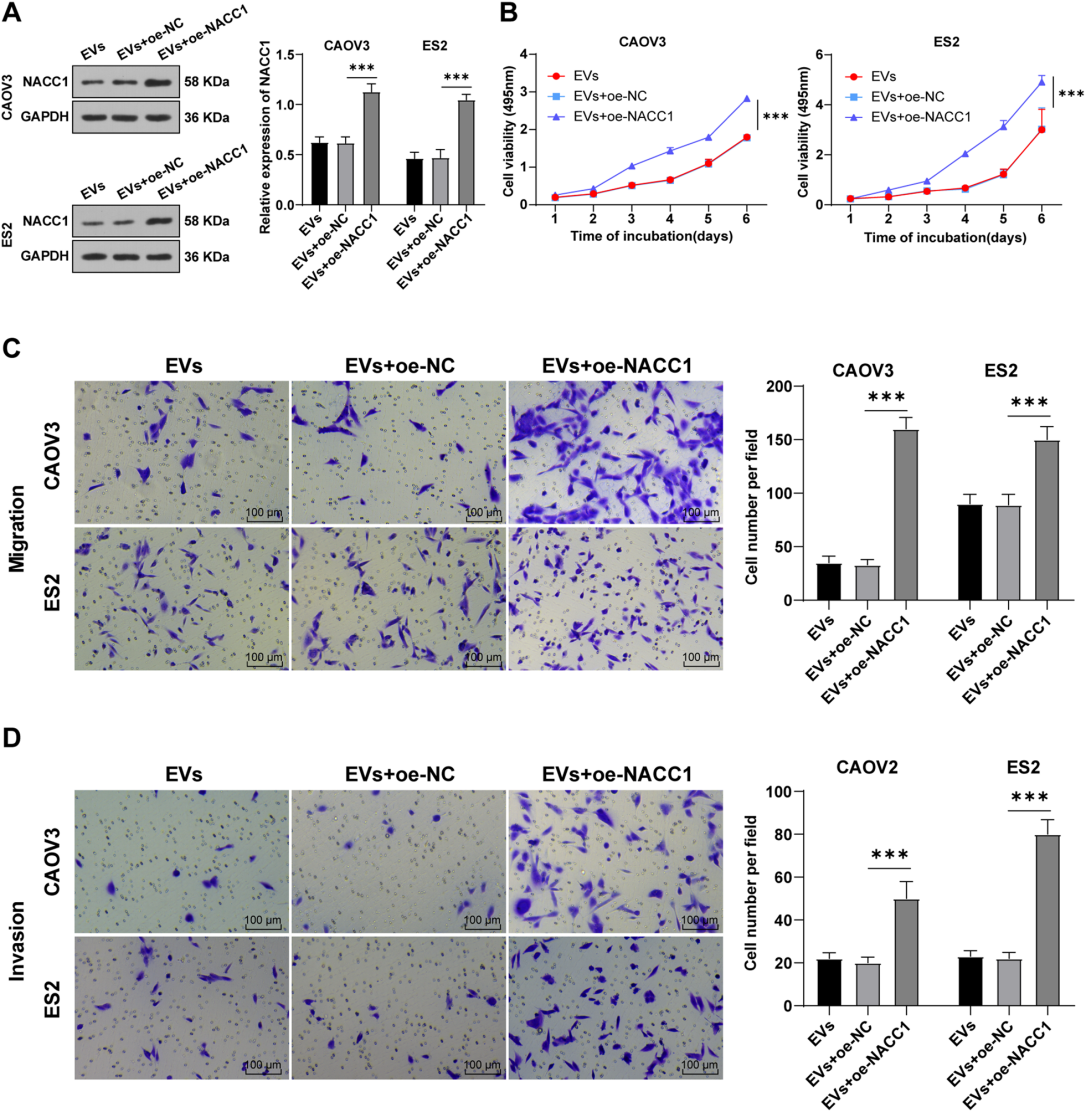
NACC1 overexpression reversed the inhibition of hMSC-EVs on OC cell proliferation, migration and invasion. NACC1 overexpression was accomplished in EVs-treated CAOV3/ES2 cells. **A** WB was used to detect the expression of NACC1; **B** MTT assay was adopted to assess cell viability; **C**, **D** Transwell assays were performed to detect OC cell migration and invasion. Cell experiments were replicated 3 times. Data were showed as mean ± SD. One-way ANOVA was performed for comparisons between groups and Tukey's multiple comparisons test was adopted for the post hoc test. ***P < 0.001

NACC1 was overexpressed in EVs-treated CAOV3/ES2 cells to investigate the role of NACC1 in OC cells. First, WB detection revealed markedly elevated NACC1 in CAOV3 and ES2 cells (all *P* < 0.05) (Fig. 6A). Thereafter, we found that the inhibitory effect of hMSC-EVs on OC cells was averted after NACC1 overexpression (all *P* < 0.01) (Fig. 6B–D). Conjointly, hMSC-EVs regulated NACC1 expression by carrying miR-18a-5p into OC cells, thereby inhibiting the proliferation, migration, and invasion of OC cells.

### NACC1 promoted the AKT/mTOR pathway activation in OC cells

**Fig. 7:**
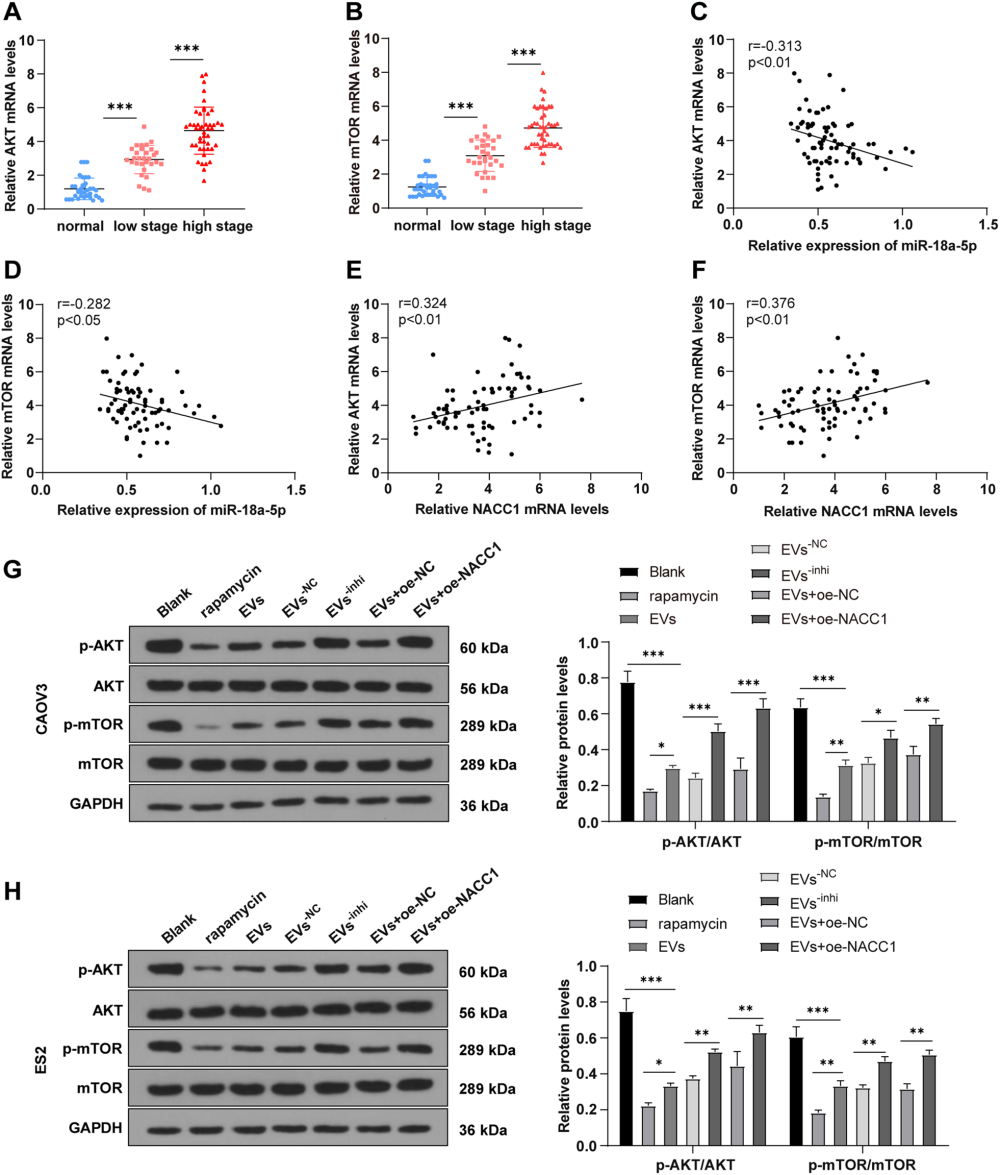
NACC1 activated the AKT/mTOR pathway in OC cells. **A**, **B** RT-qPCR was used to detect the expression of AKT and mTOR in OC tissues and benign ovarian tumor tissues; **C**–**F** Pearson analysis was used to assess the correlation of miR-18a-5p/NACC1 with the AKT/mTOR pathway; **G**, **H** WB was employed to measure the levels of p-AKT, AKT, p-mTOR, and mTOR in cells. Cell experiments were repeated 3 times. Data were expressed as mean ± SD. One-way ANOVA was adopted for comparisons between groups and Tukey's multiple comparisons test was performed for the post hoc test. *P < 0.05,**P < 0.01, ***P < 0.001

To further study NACC1 downstream mechanism in OC, we explored the downstream regulatory signaling pathway. RNA-binding motif protein 11 is documented to be highly expressed in OC tissues and positively regulates the activation of AKT/mTOR pathway [42], indicative of the significance of AKT/mTOR pathway in OC. Hence, we hypothesized that highly-expressed NACC1 in OC mediated AKT/mTOR expression. We first found that AKT and mTOR were highly expressed in OC tissues by RT-qPCR, and their levels were gradually increased with tumor progression (all *P* < 0.05) (Fig. 7A, B). Pearson correlation analysis showed that the activation of AKT/mTOR pathway was significantly negatively correlated with miR-18a-5p expression and positively correlated with NACC1 expression (all *P* < 0.05) (Fig. 7C–F).

To further investigate the effect of NACC1 on AKT/mTOR pathway, CAVO3/ES2 cells were treated with rapamycin and different EVs, and the overexpression of NACC1 was achieved in CAOV3/ES2 cells treated with EVs. The phosphorylation levels of AKT and mTOR proteins were detected by WB, which indicated significantly decreased p-AKT/AKT and p-mTOR/mTOR levels after the addition of rapamycin and EVs, but the trend of reduction was more significant in the rapamycin group. However, the levels of p-AKT/AKT and p-mTOR/mTOR were notably increased by EVs^−inhi^ treatment and also elevated after treatment of EVs and NACC1 overexpression (all *P* < 0.01) (Fig. 7G, H). Briefly, NACC1 mediated the activation of AKT/mTOR pathway.

### hMSC-EVs enhanced OC cell sensitivity to chemotherapy drugs by transferring miR-18a-5p

**Fig. 8:**
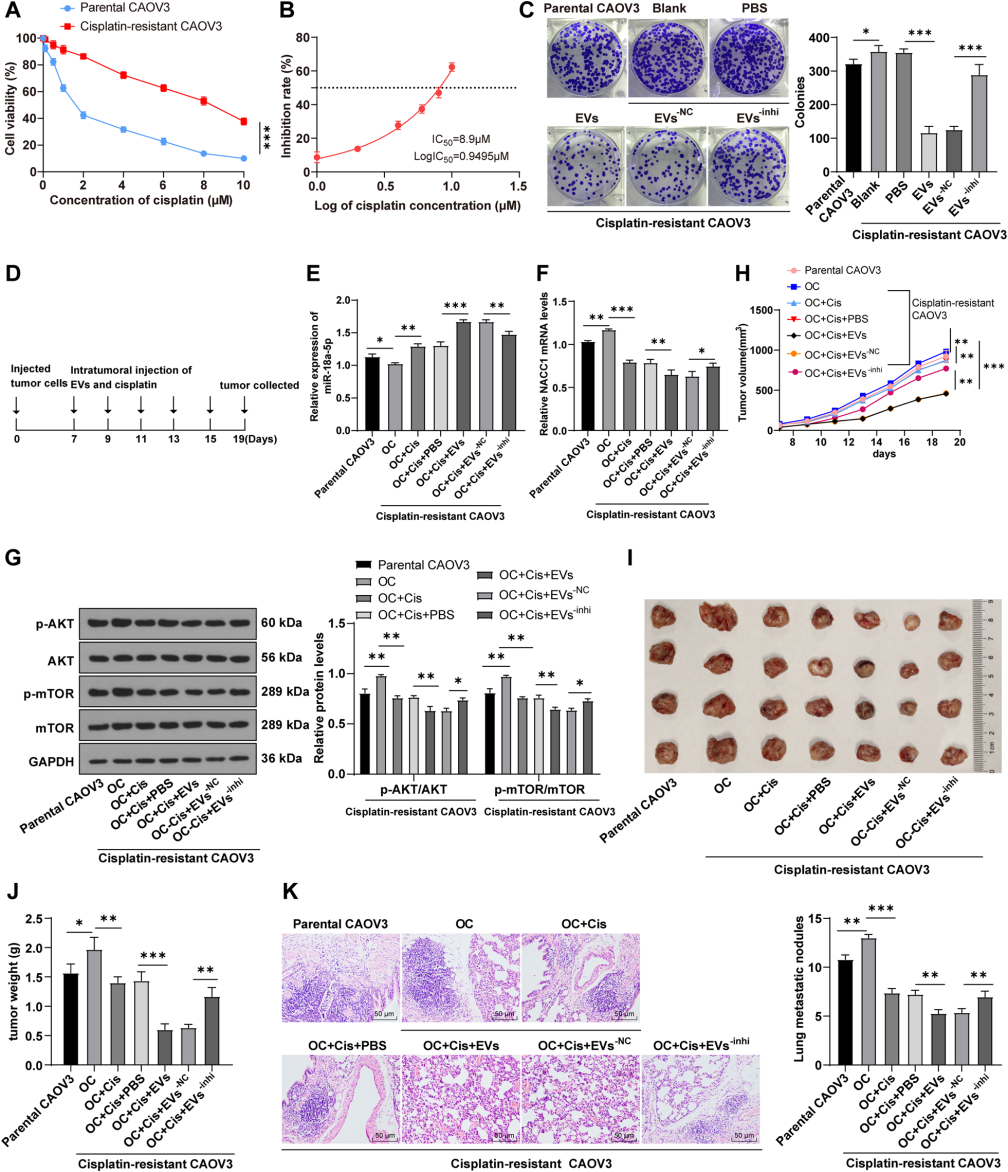
hMSC-EVs enhanced OC cell sensitivity to chemotherapy drugs by transferring miR-18a-5p. CAOV3 drug-resistant cell lines were constructed. **A** The cisplatin sensitivity of resistant cells and parental cells was compared via MTT assay; **B** The IC_50_ value of drug-resistant cells was assessed; **C** The cisplatin sensitivity of cells was compared using colony formation assay; **D** Schematic diagram of tumorigenesis test in mice; **E**, **F** RT-qPCR was used to detect the expression of miR-18a-5p and NACC1 mRNA; **G** WB was employed to measure the levels of p-AKT, AKT, p-mTOR, and mTOR in cells; **H** Tumor volume of nude mice was detected; **I**, **J** Tumors in nude mice were separated, photographed and weighed; **K** HE staining was used to detect tumor nodules in lung tissues of nude mice. Independent test was repeated 3 times. Data were presented as mean ± SD. One-way ANOVA was used for comparisons between groups and Tukey's multiple comparisons test was adopted for the post hoc test. *P < 0.05, **P < 0.01, ***P < 0.001

To investigate whether hMSC-EVs affect the cisplatin resistance of OC cells, we first constructed a CAOV3 drug-resistant cell line. The sensitivity of resistant cells and parental cells to cisplatin was compared and the results unveiled reduced sensitivity of drug-resistant cells to cisplatin (*P* < 0.05), which indicated the successful construction of drug-resistant cells (Fig. 8A). Further, the IC_50_ value of cisplatin in the OC drug-resistant cell line CAOV3 was calculated to be 8.9 μM (Fig. 8B). Moreover, we performed colony formation assay and found that the drug-resistant cells had higher colony formation number than the parental cells (*P* < 0.01). The OC drug-resistant cell line CAOV3 was added with EVs form different treatment groups and cisplatin at IC_50_ value. The results unraveled that the colony formation number was markedly decreased in the EVs group (*P* < 0.01), while increased in the EVs^−inhi^ group compared with the EVs^−NC^ group (*P* < 0.01) (Fig. 8C), suggesting that hMSC-EVs could enhance the drug sensitivity of tumor cells through miR-18-5p transmission.

Additionally, the effect of hMSC-EVs on drug sensitivity of OC cells was further verified in vivo. Nude mice were injected with parental cells and OC drug-resistant cell line CAOV3. The mice injected with drug-resistant cell line CAOV3 were intraperitoneal injected with EVs form different treatment groups and cisplatin on the 7^th^ day, once every 2 days, 5 times in total (Fig. 8D). Firstly, the levels of miR-18a-5p and NACC1 mRNA were detected by RT-qPCR and levels of p-AKT and p-mTOR were measured by WB. The results showed the drug-resistant mice injected with EVs and cisplatin exhibited significantly elevated miR-18a-5p, diminished NACC1 mRNA, p-AKT, and p-mTOR, and reduced tumorigenesis ability, tumor volume and weight (all *P* < 0.01), and decreased number of lung metastatic nodules. However, after inhibiting miR-18a-5 in EVs, miR-18a-5p was lowered, levels of NACC1 mRNA, p-AKT and p-mTOR were elevated, the ability of EVs to inhibit tumor growth was obviously weakened, the number of lung metastatic nodules was raised (all *P* < 0.01) (Fig. 8E–K). Collectively, hMSC-EVs promoted OC cell sensitivity to cisplatin by carrying miR-18a-5p.

## Discussion

OC ranks eighth in most common cancers that lead to death [43]. Previous study has proposed that MSCs are critical in suppressing OC development [44]. In addition, adults MSCs are significant tumor-targeted delivery mediators in intercellular communication via secreting EVs that contain a large amount of encapsulated miRNAs [45]. We highlighted that hMSC-EVs-transported miR-18a-5p inhibited OC malignant episode, together with the resistance to the chemotherapy drug cisplatin.

Firstly, we successfully separated and characterized normal hMSC-EVs and then observed that hMSC-EVs can be internalized by CAOV3/ES2 cells and repressed OC cell proliferation, migration, and invasion. MSC-EVs possess prominent importance in inhibiting the tumorigenesis and angiogenesis of OC [46]. Placental EVs are implicated in preventing OC cell growth [47], suggestive of the importance of hMSC-EVs in limiting OC development. miRNAs, engaged in OC development, can be used as diagnostic and prognostic biomarkers in OC [48, 49]. Subsequently, miR-18a-5p, the most possible miRNA, was screened out through the GEO database and then we detected its expression in OC, which unraveled a decreased miR-18a-5p level in tissues and reduced circulating level of miR-18a-5p in serum of OC patients. Additionally, OC patients with low miR-18a-5p expression presented short survival. Consistently, miR-18a-5p is reported to be downregulated in OC cells and tissues [36]. miR-18a exerts repressive roles in OC cells, whose low expression is linked with the poor prognosis and malignant phenotype of OC patients [50]. Interestingly, EVs-carried miRNAs display suppressive roles in OC cell proliferation and growth [51]. Next, we tested this conception in OC. RT-qPCR assay revealed an elevated miR-18a-5p in EVs and documented that miR-18a-5p was encapsulated in EVs. After EVs^−inhi^ treatment, miR-18a-5p was reduced in OC cells, thereby promoting the OC cell growth, while miR-18a-5p overexpression inhibited OC cell growth. MSCs tumor-homing ability and anti-tumor property enable them to deliver therapeutic agents to the target cancer cells, including epithelial OC [24]. MSC-EVs are believed to reduce the irritants that easily lead to cancer progression by the delivery of miRNAs [52]. miR-18a-5p is abundant in embryonic stem cell-derived small EVs [53]. miR-18a mimic could relieve OC cell migration and invasion [36]. Taken together, hMSC-EVs inhibited OC cell proliferation, migration, and invasion by carrying miR-18a-5p.

Moreover, we explored the downstream target of miR-18a-5p in OC. NACC1 was screened to be the most possible target gene using databases, which was proved to be upregulated in OC patients and cells and negatively-linked with miR-18a-5p. NACC1 elevation is related to aggressiveness and chemoresistance development in OC [54]. Consistently, NACC1 has been demonstrated to be highly-expressed in OC and its silencing causes OC cell apoptosis to increase chemotherapy sensitivity [55]. Thereafter, the binding sites of miR-18a-5p and NACC1 were anticipated by Starbase database and ascertained using dual-luciferase assay. NACC1 was diminished after EVs treatment and increased after miR-18a-5p inhibition. As far as we know, no previous research has investigated the relationship of miR-18a-5p and NACC1 in OC. Our findings initially unveiled that miR-18a-5p targeted and inhibited NACC1 in OC.

NACC1 is acknowledged to exert imperative property in chemotherapy resistance and growth of tumor cells [34, 56]. Furthermore, the role of NACC1 in OC cell growth was studied. After NACC1 overexpression in EVs-treated OC cells, the inhibition of hMSC-EVs on OC cell malignant episodes was annulled. Consistently, NACC1 restoration stimulates proliferation, migration, and invasion in OC cells [35, 57], while NACC1 knockdown possesses the contrary function [58]. Briefly, hMSC-EVs regulated NACC1 expression by carrying miR-18a-5p, thereby suppressing OC cell growth. Additionally, we discussed the downstream mechanism of NACC1 in OC. The levels of AKT and mTOR were measured, which were upregulated in OC tissues and increased with OC progression. Additionally, we observed the inverse correlation between AKT/mTOR pathway and miR-18-5p and positive correlation between AKT/mTOR pathway and NACC1. AKT/mTOR pathway is normally activate in OC and plays a critical function in proliferation, growth and metastasis [59–61]. AKT/mTOR pathway is essential and impeding this signaling is known as a therapeutic tool to enhance chemosensitivity in OC [62]. Moreover, the levels of p-AKT/AKT and p-mTOR/mTOR in OC cells were raised in OC cells, but lowered after EVs or rapamycin treatment, while elevated after NACC1 overexpression in EVs-treated OC cells or EVs^−inhi^ treatment. NACC1 activates the AKT/mTOR pathway [63], whereas NACC1 silencing inhibits AKT/mTOR pathway activation [64]. To sum up, NACC1 promoted the activation of AKT/mTOR pathway in OC.

MSCs mediate the drug resistance formation [65] and MSC-EVs are demonstrated a great promise in cancer treatment [66]. miR-18-5p is relevant to drug sensitivity [67]. Herein, we constructed the drug-resistant OC cell line and found that hMSC-EVs enhanced the sensitivity to cisplatin and suppressed the ability of tumorigenesis of OC cells through miR-18-5p transportation. The above observations are consistent with the idea that miR-18a-5p presents a neoplasm-repressive function in cisplatin-resistant OC cells [41]. Similarly, upregulation of miR-18-5p prevents tumor growth and chemoresistance of breast cancer [68]. Conjointly, hMSC-EVs inhibited OC cell cisplatin resistance and tumorigenesis by transferring miR-18a-5p.

To conclude, our study supported that hMSC-EVs-delivered miR-18a-5p repressed OC cell proliferation, migration and invasion, and enhanced cell sensitivity to cisplatin and reduced tumorigenesis in OC. However, we only investigated the downstream mechanisms of miRNAs. In addition, the role of NACC1/AKT/mTOR in the miR-18a-5p-mediated chemotherapy response lacks in vitro and in vivo validation. Further studies shall be conducted to explore the role of lncRNAs that mediate the ACC1/AKT/mTOR axis via ceRNA mechanism in OC and the function of the NACC1/AKT/mTOR pathway in miR-18a-5p-mediated chemotherapy response in OC, with the expectation to develop a promising approach for treating OC. Moreover, our results did not specifically focus on the EVs in chemoresistance, and most of our studies were based on non-resistant cells. Combining benign and tumor ovarian tissues for microarray analysis might be a better screening strategy. We shall focus on the investigation of benign and tumor ovarian tissues to find more miRNAs related to OC development. Meanwhile, the sample size shall be expanded to detect the circulating level of serum miR-18a-5p in OC patients to explore the prognostic ability of circulating miR-18a-5p level on OC, thus providing a new biomarker for the prognosis of OC. Furthermore, we should carry out studies from various aspects and perform in vivo experiments with different miRNA delivery systems.

## Supplementary Information

**Additional file 1: Table S1.** miRNAs expressed in hMSC-EVs were identified through the EVmiRNA database.

**Additional file 2: Table S2.** miRNAs in the intersection of microarray GSE161784 and EVmiRNA database were identified.

## Data Availability

The raw data supporting the conclusions of this article will be made available by the authors, without undue reservation.
